# Quantification of the Host Response Proteome after Mammalian Reovirus T1L Infection

**DOI:** 10.1371/journal.pone.0051939

**Published:** 2012-12-11

**Authors:** Alicia R. Berard, John P. Cortens, Oleg Krokhin, John A. Wilkins, Alberto Severini, Kevin M. Coombs

**Affiliations:** 1 Department of Medical Microbiology, Faculty of Medicine, University of Manitoba, Winnipeg, Manitoba, Canada; 2 Manitoba Center for Proteomics and Systems Biology, University of Manitoba, Winnipeg, Manitoba, Canada; 3 National Microbiology Laboratory, Winnipeg, Manitoba, Canada; 4 Manitoba Institute of Child Health, University of Manitoba, Winnipeg, Manitoba, Canada; Institute for Animal Health, United Kingdom

## Abstract

All viruses are dependent upon host cells for replication. Infection can induce profound changes within cells, including apoptosis, morphological changes, and activation of signaling pathways. Many of these alterations have been analyzed by gene arrays to measure the cellular “transcriptome.” We used SILAC (stable isotope labeling by amino acids in cell culture), combined with high-throughput 2-D HPLC/mass spectrometry, to determine relative quantitative differences in host proteins at 6 and 24 hours after infecting HEK293 cells with reovirus serotype 1 Lang (T1L). 3,076 host proteins were detected at 6hpi, of which 132 and 68 proteins were significantly up or down regulated, respectively. 2,992 cellular proteins, of which 104 and 49 were up or down regulated, respectively, were identified at 24hpi. IPA and DAVID analyses indicated proteins involved in cell death, cell growth factors, oxygen transport, cell structure organization and inflammatory defense response to virus were up-regulated, whereas proteins involved in apoptosis, isomerase activity, and metabolism were down-regulated. These proteins and pathways may be suitable targets for intervention to either attenuate virus infection or enhance oncolytic potential.

## Introduction

An understanding of how host cells respond to an invading pathogen may provide important clues about how to either attenuate pathogenesis mediated by the agent, or may facilitate attempts to subvert the pathogen into a ‘beneficial’ organism. At any given time, a cell's genome remains constant. However, during a cell's life cycle, proteins that are expressed within the cell (proteome) will vary depending on numerous external influences that alter genomic biochemical interactions. A cell's proteome is dependent on the location of the cell, different stages of its life cycle or environmental conditions. When a cell becomes exposed to an invading microorganism, such as a virus that requires the host cell's machinery and metabolism to replicate, the cell's proteome reflects the specific alterations of the pathways induced by infection.

Previous analyses of how cells respond to various stimuli have used microarray technologies which measure the cellular “transcriptome” [1], [2]. For example, tomato mRNAs are differentially expressed at different times post infection by Cucumber mosaic virus [3]. In addition, microarray analysis of host genetic expression profiles in different HIV disease progression patients detected differences in host responses that may influence severity of the disease [4]. However, there is frequently little concordance between microarray and protein data [5], [6], partly because mRNA levels cannot provide information about protein post-translational modifications that may prevent or initiate protein activation or degradation. In addition, genes may also encode more than one protein. Therefore, monitoring a cells' proteome should provide additional insight into host responses during infection.

Recent advances in mass spectrometry and bioinformatics have now provided several general ways that allow in-depth quantitative analysis of large numbers of proteins, and these are being used in several systems to examine the proteome. These methods include 2D difference gel electrophoresis (2D DIGE), isotope-coded affinity tagging (ICAT) and the similar iTRAQ (Isobaric tag for relative and absolute quantitation), as well as stable isotope labeling by amino acids in cell culture (SILAC) [7], [8]. We elected to use SILAC, which involves labeling two separate cultures, one light (L) and one heavy (H), with isotopically differentiated essential amino acids [9]. An advantage with SILAC is the early mixing of samples, which reduces sample-to-sample variability. In addition, several comparative studies have suggested that non-gel based methods such as SILAC can identify many more regulated proteins than gel-based studies (reviewed in [10]). SILAC has been recently used in virus studies to explore the secretory pathway of host cells in coronavirus replication [11], and examining A549 cell responses after infection by either respiratory syncytial virus [12], or influenza virus [13].

The mammalian reoviruses (MRV) are the prototype viruses in the family Reoviridae. This family currently contains 12 genera [14], many of which, unlike the prototype virus, are highly pathogenic in their hosts. MRV are non-enveloped viruses with genomes of 10 segments of double-stranded (ds) RNA surrounded by 2 concentric protein capsids (for detailed reviews see [15], [16]). Numerous stages in the MRV life cycle involve interaction of viral components with host proteins. MRV replication is primarily cytoplasmic, starting with binding of the virion to host receptors and receptor-mediated endocytosis. The virion is converted to intermediate subviral particles by proteolytic cleavage of the outer capsid proteins by host proteases. Once cleavage occurs, the virion enters the cell's cytosol and the released core particles act as transcriptionally active RNA-dependent RNA polymerase complexes to produce viral mRNA. The viral mRNA molecules are translated by host ribosomes to produce viral proteins. Virus proteins coalesce into non-membrane inclusions in the cytoplasm by an unknown mechanism. Interactions with host interferon-regulated proteins, such as RNase-L and PKR aid to switch production of transcripts needed for replication purposes to production of progeny virions. Recoating occurs inside the cell producing infectious intracellular virions, which are released during cell lysis. Because of these viral-host interactions, it is expected that infection of the cell with reovirus will cause differential regulation of many host proteins. MRV affect the gastrointestinal system or cause respiratory infections in humans and recent work has shown that MRV may be a useful oncolytic agent [17], [18]. Most studies have shown that MRV strain T3D has oncolytic properties, and this subtype has been used in clinical studies [19], [20]. However, it has recently been shown that all of the reovirus subtypes show oncolytic potential [17]. Thus, a better understanding of host responses to MRV infection is needed. Previous gene array analyses showed that MRV infection induces transcription of genes involved in apoptosis and DNA repair, as well as alterations in the expression of genes involved in host cell cycle regulation [21], [22]. A recent gel-based proteomic screen of MRV-infected murine myocytes demonstrated regulation of several proteins, including heat shock proteins and interferon-response proteins [23].

In this study we used SILAC to examine host protein responses in human embryonic kidney (HEK)-293 cells after 6 and 24 hours of infection by MRV strain T1L. The two time points were chosen to represent an early time point when viral particles are being assembled and a later time point when the mature virus particles are starting to be released. These time points in the viral life cycle are therefore potentially important in host cell and virus interactions that would be represented by differences in host protein abundances in the cell. We identified thousands of proteins at both time points, and a small percentage of these proteins were significantly differentially regulated at each time point. From these data, biological functions and network analysis were performed using DAVID bioinformatics resource [24], [25], as well as Ingenuity Pathway Analysis (Ingenuity® Systems, www.ingenuity.com). These pathway analyses indicated that cell death, cell growth and proliferation, molecular transport, gene expression, and inflammatory response pathways are most affected by MRV infection.

## Methods

### Cells and viruses

#### Viruses

Reovirus serotype 1 Lang (T1L) and serotype 3 Dearing, Cashdollar strain (T3D) are laboratory stocks. The viruses were grown and titrated in mouse L929 cell monolayers in Joklik's Suspension Modified Minimal Essential Medium (J-MEM) (GIBCO products, Grand Island, NY, USA) supplemented with 5% fetal bovine serum (FBS), and 200 mM l-glutamine, as previously described [26].

#### Cells

All cell lines that were used, HEK293, L929, A549, CaCo2 and HeLa cells, were acquired from ATCC, catalogue numbers CRL-1573, CCL-1, CCL-185, HTB-37 and CCL-2, respectively. For SILAC preparation, HEK293 (Human embryonic kidney) cells were grown in D-MEM (Dulbecco's Modified Eagle Medium) supplied in a SILAC^TM^ Phosphoprotein Identification and Quantification Kit (Invitrogen Canada Inc.; Burlington, Ontario). D-MEM was supplemented to contain 10% dialyzed FBS, and either 100 mg/L ‘Light’ (normal) lysine and arginine or ‘Heavy’ (^13^C_6_-lysine and ^13^C_6_-/^15^N_4_-arginine). One set of HEK293 cells were allowed to double 7 times in L media and another set of HEK293 cells were allowed to double 7 times in H media. Three biologic replicates were performed.

#### SILAC Infection

Approximately 10^7^ L-labeled cells in T75 flasks were infected with T1L at a multiplicity of infection (MOI) of 5 PFU per HEK293 cell. Equivalent numbers of H cells were mock-infected as control. The flasks were incubated at 4°C for one hour, with gentle rocking every 10–15 minutes, to allow virus to adsorb and to synchronize infections. An overlay of 10 mls of pre-warmed appropriate L or H media was then added to each flask, and the infected cell cultures were incubated at 37°C for 6 and 24 hours.

### Titration

The titers of samples were determined by plaque assays as previously described [27]. Briefly, samples were diluted and used to infect 12 well plates of L929 cells, then overlaid with a nutrient agar solution. After plaque development, cells were stained with crystal violet, and plaques were counted to determine viral titers.

### Photomicrography

Infected and mock-infected cells in T25 flasks, and 12 well plates, were examined microscopically for cytopathic effect (CPE) at 0, 3, 6, 9, 12, 15, 18, 24, 30, 36, 48 and 72 hours post infection with a Nikon TE-2000, and cells were photographed with a Canon- A700 digital camera. Images were imported into PowerPoint and slight adjustments made in brightness and contrast to the exact same degree for all of the pictures, which did not alter image context with respect to each other.

### Cell Viability

HEK293 and L929 cells were infected with either T1L or T3Dc, mock infected, or treated with 100 μg/ml puromycin to act as a positive control. At 0, 3, 6, 9, 12, 15, 18, 24, 30, 36, 48 and 72 hours post infection, cells were harvested, and aliquots were combined with Trypan Blue solution (Sigma, cat#T8154-100ML) at 1 1 ratio. A hemocytometer was used to count a total of 200 cells, as well as all dead cells, in each sample.

### Immunofluorescence Microscopy

HEK293 cells were mounted on autoclaved 12-spot slides, letting the cells adhere to the slides overnight at 37°C. Once adhered, the spots were washed two times with 1X PBS. The cells were counted and virus was added to each spot at an MOI of 5 in serum free media. The virus was allowed to adsorb on the cells for one hour on ice, to ensure synchronization of the infection, then complete media was used for overlay. The spots were incubated for 0, 4, 8, 12, 18 and 24 hours at 37°C. At each time point, spots were washed (3×) with PBS and fixed with paraformaldehyde (∼15 mins).

Once fixed, all spots were washed and cells were permeabilized with 0.2% TritonX-100 in PBS for 5 mins. Spots were blocked using PBS with 1% BSA and 5% FBS, followed with primary antibody (in-house rabbit anti-reovirus), then with Alexa Fluor® 488 Goat anti-Rabbit (Invitrogen, cat#A11008) secondary antibody, DAPI (Invitrogen, cat#D1306), and Alexa Fluor® 546 Phalloidin (Invitrogen, cat#A22283). Anti-fade reagent (Invitrogen, cat#P36935) was added to each spot before slides were covered with coverslips. Slides were examined on a Zeiss Axio Observer Z1 inverted microscope using 20× objective and fluorescence illumination using Exfo Xcite. Images were acquired using AxioVision 4.8.2 software.

### Cell Fractionation

At 6 and 24 hours post infection (hpi), both L infected and H mock-infected cells were collected and counted. Aliquots of cultures were also saved for virus titration to confirm infection status. Equal numbers of L and H cells were mixed, and mixed cells were washed three times with ice-cold Phosphate Buffered Saline (PBS). The washed cells were re-suspended in NP-40 buffer (140 mM NaCl, 1.5 mM MgCl2, 10 mM Tris [pH 7.4], 0.5% NP-40) at a concentration of 2.0×10^8^ cells/ml, with the addition of Pepstatin A to a concentration of 1.1 µM.

The mixed, washed cells were then incubated on ice for 30 minutes with gentle mixing after 15 minutes. The nuclei were pelleted by centrifuging at 300xg for 10 minutes. The cytosol (supernatant) was transferred to a fresh microfuge tube; and the two fractions (nuclear pellet and cytosol) were frozen at −80°C until further processing took place.

### Reduction and Alkylation

Protein concentrations in cytosolic lysates were determined using a BCA^TM^ Protein Assay Kit (Pierce; Rockford, IL). Aliquots corresponding to 300 µg of protein were diluted 6-fold with 100 mM ammonium bicarbonate. Samples were reduced with 10 mM (final concentration) of dithiothreitol (DTT) for 45 minutes at 60°C, alkylated with 50 mM of iodoacetic acid for 30 min at room temperature in the dark. Excess alkylating agent was removed by addition of 16 mM DTT. Samples were digested with 6 μg of sequencing grade trypsin (Promega, cat#V5111) overnight at 37°C. A different protein standard was used in the first experiment compared to the second and third experiments, which resulted in less protein being processed, loaded and identified in the second and third experiments.

### First dimension fractionation

An Agilent 1100 Series HPLC system with UV detection at 214 nm was used for off-line first dimension fractionation. 100 μg of each digested protein sample was loaded onto a 1×100 mm XTerra C18 column (Waters, Milford, MA) and separated using a linear 0–35% water-acetonitrile gradient in 60 minutes at 150 μL/min flow rate. Both eluents contained 20 mM ammonium formate pH 10 [28], [29]. Sixty fractions were collected and concatenated pair-wise according to previously described procedures [28]. Briefly, 60 one-minute fractions were collected, and the first 30 fractions were combined with the second 30 fractions (for example, fraction 1 with fraction 31; fraction 2 plus fraction 32, etc). The 30 concatenated fractions were lyophilized, and stored at −80°C until second dimension LC-ESI/MS was performed.

### Second dimension Liquid chromatography – Electrospray Mass Spectrometry

The 30 concatenated fractions collected from the first dimension high pH reversed-phase (RP) fractionations were analyzed separately by low-pH RP LC with on-line ESI/TOF Quadupole MS/MS detection (QStar Elite) as was previously described [13]. Raw MS/MS data were analyzed using the Protein Pilot^TM^ (ABSciex) program that identifies proteins based on cumulative peptide scores, and generates Light:Heavy ratio scores based on intensity ratios of corresponding peptide peaks.

### Protein quantitation

Within each experiment, proteins identified with L:H ratios were normalized using z-score analysis, described previously [13]. Briefly, all L:H ratios were converted to log_2_, and average ratios and standard deviations were determined after removal of significant outliers. Each protein's ratio was then converted to a z-score using the formula:
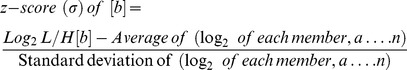
where b represents a single protein in the dataset. The z-score measures standard deviation units (σ) a ratio is from the mean. For example, a z-score of >1.960σ or <−1.960σ indicates that it is outside the 95% confidence level, >2.567σ or <−2.576σ indicates 99% confidence, and >3.291σ or <−3.291σ indicates 99.9% confidence.

### DAVID analysis

#### Functional Annotation Tools

DAVID analysis was performed using lists of proteins up and down regulated previously generated from Protein Pilot analysis [24], [25]. Proteins determined differentially regulated by statistical analysis were tabulated in Excel and their GI accession numbers were uploaded into DAVID for functional annotation tool analysis. DAVID analysis enabled enrichment of functional-related gene groups, cluster redundant annotation terms and visualization of BioCarta & KEGG pathway maps that may play a role in the dataset uploaded.

### Ingenuity Pathway Analysis

#### Network Generation

Data sets derived from the Protein Pilot analysis, containing gene identifiers and corresponding expression values, were uploaded into the Ingenuity® Systems application. Each gene identifier was mapped to its corresponding gene object in the Ingenuity Pathways Knowledge Base. A z-score cutoff of ±3.29, ±2.58 and ±1.96 (representing 99.9%, 99% and 95% confidence scores, respectively) was set to identify proteins whose expression was significantly differentially regulated. These proteins, called focus proteins, were overlaid onto a global molecular network developed from information contained in the Ingenuity Pathways Knowledge Base. Networks of these focus genes were then algorithmically generated based on their connectivity.

#### Functional Analysis of a Network

The Functional Analysis of a network identified the biological functions and/or diseases that were most significant to the genes in the network. The network genes associated with biological functions and/or diseases in the Ingenuity Pathways Knowledge Base were considered for the analysis. Fischer's exact test was used to calculate a p-value determining the probability that each biological function and/or disease assigned to that network was due to chance alone.

### Immunoblotting

Western blot analysis of infected HEK 293 cells was performed as described previously [13]. Briefly, at 6 and 24 hours post infection with T1L reovirus at MOI 5, unlabelled HEK293 cells were harvested similar to the cell preparation (above). Cytosolic proteins were resolved on 8 W×6.5 H×0.1 cm 10% SDS-PAGE gel at 120V for 70 min. Proteins were then transferred to a polyvinylidene difluoride (PVDF) membrane at 100V for 60 min, and the transfer was confirmed using Ponceau staining. The membranes were blocked using 5% skim milk in TBST and probed using various antibodies in 1% BSA in TBST. Primary antibodies were in-house rabbit anti-reovirus, rabbit anti-lactotransferrin (Millipore, cat#07-685), α-ISG15 (Rockland, cat#200-401-438), α-SCG2 (Abnova cat#PAB17107), α-hnRNPA1 (Cell Signaling, cat#8443), GAPDH (Cell Signaling, cat#2118), α-OAS3 (Abcam, cat#ab71780), α-IFIT2 (Abcam, cat#ab55837), and α-PTPN12 (Abcam cat#ab90641), and mouse anti-STAT1 (Cell Signaling, cat#9176) and α-BAD (Santa Cruz, cat#sc-8044). The secondary antibodies were the appropriate horseradish peroxidase (HRP)-conjugated rabbit anti-mouse or goat anti-rabbit (Cell Signaling, cat#7076 and cat#7074, respectively). Bands were detected by enhanced chemiluminescence using an Alpha Innotech FluorChemQ MultiImage III instrument.

## Results

### Reovirus successfully infects HEK293 cells

**Figure 1:**
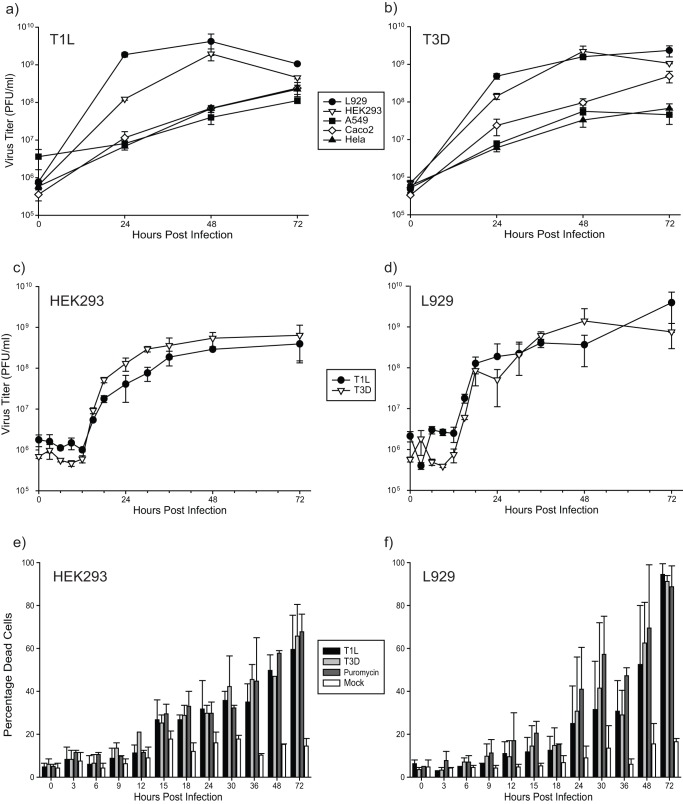
Kinetics of reovirus growth and viral-induced cytopathology. Each of five different cell lines (L929, A549, HEK293, CaCo_2_ and Hela) were infected at MOI  = 1 PFU/cell with T1L (a) or T3D (b). Cell lysates were harvested at 0, 24, 48 and 72hpi and titrated. Experiments were performed in triplicate; error bars represent standard error. Virus titers were greatest in the L929 and HEK293 cells for both virus strains. HEK293 (c) and L929 (d) cells were then re-analyzed as in (a) and (b) after infection at MOI  = 5 and at additional time points. Aliquots of the infections in (c) and (d) were also assessed for cell viability by trypan blue exclusion (e and f, respectively), with 100 μg/ml puromycin used as a positive cell killing control. Experiments were performed in duplicate; error bars represent standard error.

To determine which virus subtype and cell line to use for proteomic analyses, we performed an initial growth assay of both MRV strains T1L and T3D in five different cell lines; mouse L929 which are usually used for reovirus work, and in human A549, HEK293, CaCo_2_ and Hela cells (Figure 1a&b). The cell lines that generated the highest virus titers were L929 and HEK293. Reovirus infection in L929 cells is well characterized [30]. However, reovirus growth has also been shown to be dependent on MOI and on cell type [31], [32]. We therefore performed a more extensive growth analysis of both T1L and T3D in the L929 and 293 cells (Figure 1c&d). This assay showed virus titers of both strains began to increase at 12 hours post infection (hpi) in both cell types, that growth was exponential during the next 6–12 hours, and that both strain's titers continued to increase up to about 48 hpi and then plateau after this time point.

Cell viability was also measured by Trypan Blue exclusion in both HEK293 (Figure 1e) and L929 cells (Figure 1f). The percentage of dead HEK293 cells continually increased from about 15hpi to 72hpi, and reached approximately 65% mortality irrespective of virus types. This is a lower level of cell death compared to the L929 cells, which reached more than 90% mortality by 72hpi, indicating HEK293 cells are either more resistant to reovirus induced apoptosis or the cells are slower to respond to reovirus infection.

Because HEK293 cells are human and therefore potentially more relevant, we decided to use these cell lines in subsequent experiments. Many reovirus studies have been performed with both T1L and T3D, and although most oncolytic studies have focused on strain T3D, a recent study indicates that T1L may also have oncolytic relevance [17]. Both virus strains generated similar final titers by 48 hpi, which is within the time frame we elected for SILAC analysis. Thus, with the potential to gain new insight in T1L infections, and because T1L appeared to grow efficiently in 293 cells, we decided to use this strain for subsequent analyses.

**Figure 2:**
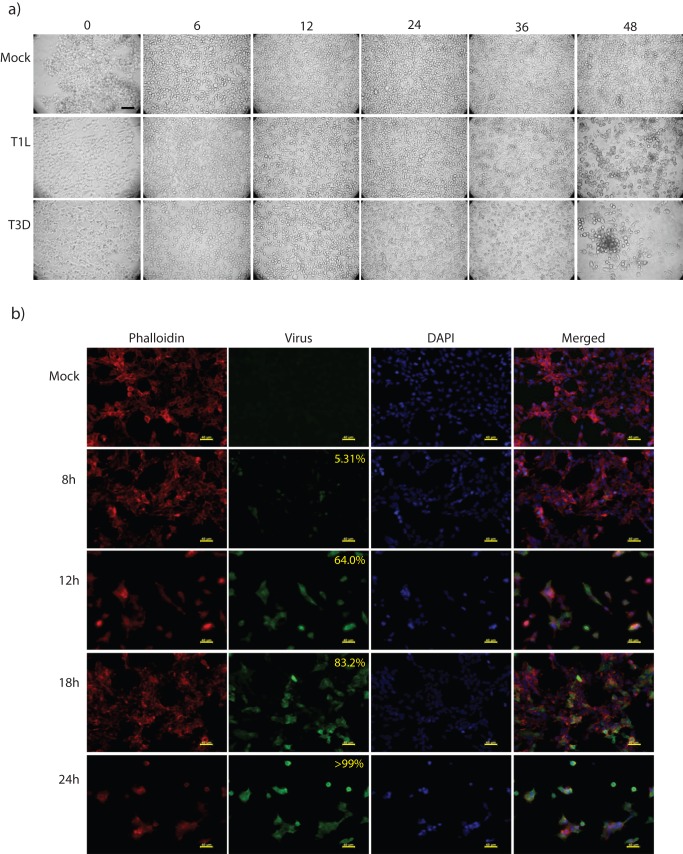
Microscopic evaluation of HEK293 cells infected with reovirus at MOI 5. (a) photomicrographs of 293 cells mock-infected (top row), or infected with T1L (middle row) or T3D (bottom row) for various times (indicated at top). Scale bar  = 40 μm. (b) HEK293 cells were infected with MRV strain T1L at MOI 5. Cells were stained for f-actin (red), reovirus (green), and DAPI (blue). At 8, 12, 18 and 24 hpi, 5.31%, 64.0%, 83.2% and 100% of cells were infected, respectively (indicated in upper rightmost corner of each image). Scale bar  = 40 μm.

Cytopathic effects in HEK293 cells were examined microscopically (Figure 2a). Little CPE was apparent by 24hpi, and only in cultures infected with the more pathogenic T3D strain. CPE was not visible in the T1L-infected cultures until 36hpi, and CPE was strongly apparent in both infected cultures by 48hpi. These results are comparable to those obtained by Trypan Blue exclusion, although Trypan Blue exclusion appeared to show some CPE occurring slightly earlier than observed microscopically. Taking into account the growth curves and cell viability of the HEK 293 cells when infected with the T1L reovirus strain, we decided to perform our SILAC proteomic analysis at an early time of infection (6hpi) before the onset of CPE and virus growth, and a later time point (24hpi) when the cells show minimal CPE and are producing a large amount of virus. These two time points should give a good indication of what is going on in the cell when the virus is first entering and replicating, then later on, when the virus is being packaged and released.

To ensure that an MOI of 5 T1L would infect the majority of HEK 293 cells, and that virus proteins were being expressed to affect host processes by the later chosen time point, immunofluorescence microscopy was performed at 8, 12, 18 and 24 hpi. Almost ⅔ of the cells demonstrated infection by 12 hpi and >99% of the cells showed virus infection by 24 hpi (Figure 2b). Previous studies indicated that increasing the MOI exponentially causes virus replication to start earlier, but the same proportion of cells become infected [30]. For our proteomic analyses, we used an MOI of 5 to ensure that by 24hpi the vast majority of cells are infected and also allowed a greater window of opportunity to observe the earlier infection process before virus production occurs (6hpi).

### Protein identification

**Figure 3:**
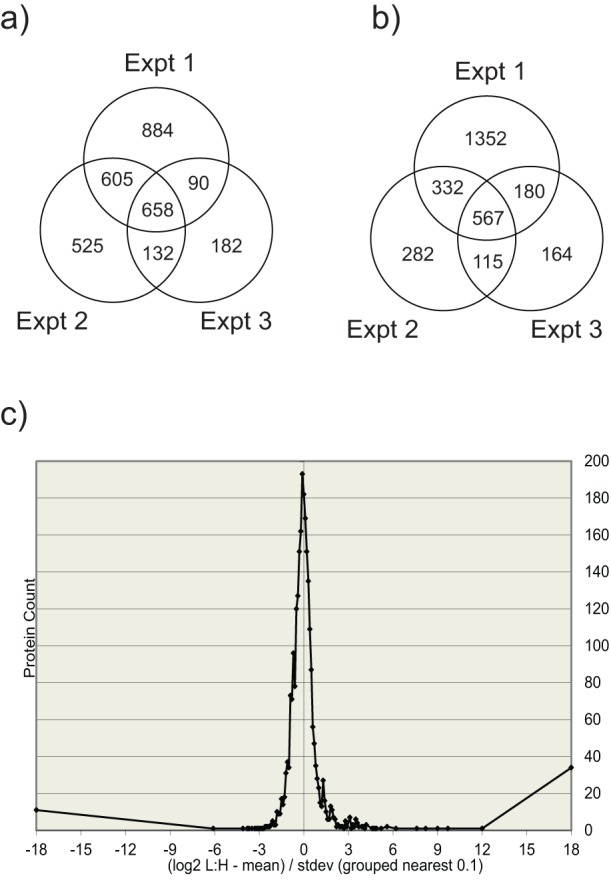
Identified protein analysis. Venn diagram summaries of the three separate T1L reovirus SILAC experiments at (a) 6hpi, when a total of 3076 unique proteins were identified and at (b) 24hpi where a total of 2992 unique proteins were identified. Overlapping numbers represent those proteins identified in more than one biological replicate (c) Population distribution represented by the log_2_ of L:H ratios of identified proteins plotted against the protein counts. This population distribution is used to determine the z-scores indicating those proteins considered significantly up or down regulated. Most proteins are seen at a 1∶1 ratio after infection, indicated by the peak of the population distribution indicated at 0. For clarity, only experiment 1 at 24hpi is shown, however population distributions were generated at both time points, for all biological replicates.

We quantitatively examined protein alterations in HEK293 cells at early (6 hpi) and late (24hpi) times after infection with MRV T1L. We performed three separate biological replicates of the infection for both time points. 2237, 1920 and 1062 proteins were identified in three experiments at the 6 h time point, with a total of 3076 unique identified proteins (Figure 3a). Of these, 1485 (48.3%) were identified in two or more experiments. After 24 hours of infection, 2431, 1296 and 1026 proteins were identified, with a total of 2992 unique species and 1194 (39.9%) found in two or more replicates (Figure 3b). More proteins were identified in the first experiment (at both time points) because, as indicated earlier, of greater sample loading. Although lower sample loading led to a decrease in the numbers of proteins identified in the second and third replicates, it had no influence on the L:H ratios that were observed.

### Protein quantitation and statistical analysis

To facilitate inter-experiment comparisons, each protein's L:H ratio was converted into log_2_ space. These log ratios were plotted in a population distribution graph and used to convert every L:H ratio into a z-score that measures how far each particular protein's L:H ratio lies in relation to the population mean and standard deviation. A representation of this for experiment 1 at 24hpi is shown in Figure 3c. Other population distributions were similar (data not shown). The population distribution-based z-scores enable the direct comparison of proteins from different experimental runs. Different confidence level cutoffs were applied to the data by z-score analysis to indicate which proteins were considered significantly differentially regulated. We used 95%, 99% and 99.9% cutoffs, which correspond to z-scores of ±1.96, ±2.8 and ±3.3, respectively. 200 proteins were observed differentially regulated in at least one experiment at 6 hpi (132 up regulated and 68 down regulated) at 95% confidence level, and 153 proteins were differentially regulated at 24hpi (104 up and 49 down regulated). Some differentially regulated proteins were observed in more than one experiment, whereas some were only identified once. Up-regulated or down-regulated proteins found in more than one experiment are listed in Table 1 and Table 2, respectively, with their corresponding average L:H score. A complete list of all differentially regulated proteins identified, including those observed in only one experiment, as well as the z-scores determined from each experiment, and how many independent screening rounds each protein ratio represents, are indicated in Supplementary Tables S1 and S2. Of those protein identified in all three experiments, ISG15, lactotransferrin and serum albumin were observed significantly up-regulated in all three experiments at 24hpi. We have used label swapping in similar experiments with other virus-cell systems (Coombs, submitted). In experiments in which infected samples are L-labeled, as here, albumin keratins, and lactotransferrin are also consistently found highly up-regulated; however, in reciprocal labelling, they appear to be highly down-regulated. In the current study, these proteins probably represent contaminants because they are consistently up-regulated after infection (a large L:H ratio) because they are naturally found in the environment in the light isotopic form, which, in the current study, corresponds to the infected sample. Because it may have antiviral properties, a western blot was performed on lactotransferrin (below) to detect whether the up-regulation observed was true or due to contamination. Other significantly up-regulated proteins include STAT1, identified in all three experiments at 24hpi, significantly up-regulated in two of the three experiments, and slightly up-regulated in the third. There were also numerous proteins, such as SCG2, IFIT2, OAS3 and BAD, that were identified in only two experiments but were significantly up-regulated in both experiments. In our study, proteins are considered up or down regulated if at least half of the biologic z-score values are ≥1.960σ and there were no major disagreements between biological replicates. More focus was placed on those proteins identified in more than one experiment; however, proteins identified in only one experiment were also used to analyze infection-induced alterations in host pathways. Numerous proteins involved in the apoptotic pathway were differentially regulated, correlating with previous findings [21], [22] as well as the virus' observed cytopathic effect.

**Table 1:** HEK293 proteins increased >95% confidence^a^.

Accession	HGNC	Name	6h L:H ratio^b^	24h L:H ratio^b^
*Proteins measured in >1 experiment (at a single time point)*				
34416	LTF	Lactotransferrin	**8.88**	**8.72**
6013427	ALBU	serum albumin precursor	**5.85**	**5.02**
17318569	K2C1	keratin 1	**4.80**	**38.35**
1683637	BAD	Bcl-2 binding component 6	**3.50**	0.76
25058739	ALB	ALB protein	**3.00**	
9963783	Q9HBZ9	RNA helicase	**2.71**	**2.51**
4557325	APOE	apolipoprotein E precursor	**2.68**	**6.48**
6563288	UBQL2	ubiquitin-like product Chap1/Dsk2	**2.67**	1.00
16553095	ASPC1	unnamed protein product	**2.39**	**2.41**
56204388	MEA1	male-enhanced antigen	**2.27**	0.88
20336350	HCD2	endoplasmic reticulum-associated amyloid beta peptide-binding protein	**2.22**	**1.86**
4885217	XPF	excision repair cross-complementing rodent repair deficiency, complementation group 4	**2.08**	
55959029	UBE2T	ubiquitin conjugating enzyme	**2.03**	1.19
22671717	HBA2	hemoglobin alpha-2	**2.02**	**3.58**
55660909	SC23B	Sec23 homolog B (S. cerevisiae)	**2.00**	1.21
29792115	SMAD3	SMAD, mothers against DPP homolog 3 (Drosophila)	**1.99**	**2.01**
48734878	Q6IPH7	RPL14 protein	**1.97**	**1.85**
7020506	UB2R2	unnamed protein product	**1.96**	1.08
4505571	SQSTM	sequestosome 1	**1.84**	1.39
57208697	REPS1	RALBP1 associated Eps domain containing 1	**1.77**	**1.70**
14042287	NRBP	unnamed protein product	**1.74**	1.02
4589628	PALLD	KIAA0992 protein	**1.73**	
55962212	CTBP2	C-terminal binding protein 2	**1.72**	0.72
55749758	DIP2B	DIP2 disco-interacting protein 2 homolog B	**1.64**	1.36
5803123	PSMF1	proteasome inhibitor subunit 1 isoform 1	1.64	**1.56**
21961219	DNJB4	DnaJ (Hsp40) homolog, subfamily B, member 4	**1.64**	0.88
12654999	DPOA2	Polymerase (DNA directed), alpha 2 (70kD subunit)	**1.63**	0.94
13543689	SLD5	GINS complex subunit 4 (Sld5 homolog)	**1.60**	
793843	RL29	ribosomal protein L29	**1.57**	**1.67**
3970842	AL1A2	RALDH2	**1.57**	1.36
56202415	STX12	syntaxin 12	**1.54**	1.18
28336	ACTB	mutant beta-actin (beta'-actin)	**1.53**	**1.46**
56205198	SNAPN	SNAP-associated protein	**1.53**	1.01
55962100	B1AN89	eukaryotic translation initiation factor 4 gamma, 3	**1.51**	0.94
13477351	CP080	Gene trap locus 3 (mouse)	**1.50**	0.95
54696534	ATPO	ATP synthase, H+ transporting, mitochondrial F1 complex, O subunit (oligomycin sensitivity conferring protein)	**1.50**	**1.59**
7705411	YTHDF2	YTH domain family, member 2	**1.47**	0.99
9623363	DPOE3	DNA polymerase epsilon p17 subunit	**1.46**	
55962775	S10AD	S100 calcium binding protein A13	**1.38**	1.34
7672784	HSP7E	heat shock protein HSP60	**1.37**	**1.75**
7576229	VIME	vimentin	**1.35**	0.99
21749793	Q8NB80	unnamed protein product	**1.31**	0.82
7705431	CCD72	coiled-coil domain containing 72	**1.30**	
48146451	DBNL	HIP-55	**1.29**	1.18
21750170	Q8NB11	unnamed protein product	**1.28**	1.29
55663125	SET	SET nuclear oncogene	**1.26**	0.95
9966827	PCNP	PEST-containing nuclear protein	**1.21**	**1.92**
6633995	SK2L2	KIAA0052 protein	1.16	**2.13**
6274552	STAT1	signal transducer and activator of transcription 1 isoform alpha	1.11	**2.38**
56203909	DDI2	DNA-damage inducible protein 2 (DDI2)	1.09	**1.90**
862457	ECHA	enoyl-CoA hydratase/3-hydroxyacyl-CoA dehydrogenase alpha-subunit of trifunctional protein	1.04	**1.81**
34532659	Q6ZTT1	unnamed protein product	1.03	**1.46**
5738608	NFU1	HIRA-interacting protein HIRIP5	1.00	**1.65**
3287825	DC1L2	Cytoplasmic dynein 1 light intermediate chain 2 (Dynein light intermediate chain 2, cytosolic) (LIC53/55) (LIC-2)	0.93	**1.56**
31652283	ACO13	thioesterase superfamily member 2	0.87	**1.65**
31880783	PELO	pelota homolog	0.77	**1.40**
18490917	SCG2	Secretogranin II (chromogranin C)		**7.62**
4731861	OAS3	2′–5′oligoadenylate synthetase 3		**5.46**
14550514	ISG15	ISG15 ubiquitin-like modifier		**4.27**
34192824	IFIT2	Interferon-induced protein with tetratricopeptide repeats 2		**3.57**
2653424	SHIP2	inositol polyphosphate 5-phosphatase		**2.44**
49574526	IFIT1	interferon-induced protein with tetratricopeptide repeats 1		**2.21**
47123412	Q6NSF2	RPLP0 protein		**1.91**
51476787	Q68D64	hypothetical protein		**1.45**
51476787	Q68D64	hypothetical protein		**1.45**

a Protein is included if at least half of the biologic z-score values are ≥1.960σ (indicated by bolding) and there are no major disagreements between biological replicates.

b L/H ratio refers to the geometric mean of all log_2_ L/H values for each given gi number, expressed as relative protein quantity in infected cultures.

**Table 2:** HEK293 proteins decreased >95% confidence^a^.

Accession	HGNC	Name	6h L:H ratio^b^	24h L:H ratio^b^
*Proteins measured in >1 experiment (at a single time point)*				
35700	PRPS2	unnamed protein product	**0.50**	**0.80**
7239177	SHPK	CARKL	**0.51**	1.08
6063691	VDAC1	porin isoform 1	**0.57**	1.14
16307253	SNP29	Synaptosomal-associated protein, 29kDa	**0.59**	**0.80**
9295343	MRRP1	HNYA	**0.61**	1.31
13112017	Q9BTT9	GYS1 protein	**0.62**	0.88
45501009	Q6NWZ1	CKAP4 protein	**0.62**	0.91
13938553	KAD6	TAF9 RNA polymerase II, TATA box binding protein (TBP)-associated factor, 32kDa	**0.63**	0.91
30354279	AKTS1	AKT1S1 protein	**0.63**	1.02
4885579	RCD1	RCD1 required for cell differentiation1 homolog	**0.64**	**0.70**
55959177	VPS45	vacuolar protein sorting 45A (yeast)	**0.64**	0.91
577315	AMPM1	KIAA0094	**0.65**	1.00
48145521	RNF14	RNF14	**0.71**	
48146107	VAMP8	VAMP5	**0.72**	1.68
7741063	NUDT4	diphosphoinositol polyphosphate phosphohydrolase type 2 beta	**0.75**	0.93
6537210	PPP6	serine/threonine protein phosphatase catalytic subunit	**0.75**	0.88
55957551	Q5TB52	3′-phosphoadenosine 5′-phosphosulfate synthase 2	**0.75**	0.88
57997480	BAG6	hypothetical protein	**0.76**	0.88
31542862	GRWD1	glutamate-rich WD repeat containing 1	**0.76**	**0.61**
50949588	LS14A	hypothetical protein	**0.78**	0.84
14041989	UBP47	unnamed protein product	**0.79**	**0.75**
51258951	XPO6	XPO6 protein	**0.79**	
5542014	DKC1	dyskerin	**0.80**	1.01
27769298	TRI25	Tripartite motif-containing 25	**0.80**	1.42
51476912	Q68D08	hypothetical protein	0.81	**0.68**
47605963	ROCK2	Rho-associated protein kinase 2 (Rho-associated, coiled-coil-containing protein kinase 2) (p164 ROCK-2) (Rho kinase 2)	0.81	**0.67**
36122	RPB2	RNA polymerase II 140 kDa subunit	0.84	**0.62**
27503838	TPPC5	Trafficking protein particle complex 5	0.85	**0.58**
15559560	BGLR	Glucuronidase, beta	0.87	**0.69**
56203612	ARFG3	OTTHUMP00000028526	0.88	**0.70**
50415798	Q6DC98	LMNB1 protein	0.89	**0.53**
14124914	GLYC	Serine hydroxymethyltransferase 1 (soluble)	0.90	**0.78**
4503141	CTSC	cathepsin C isoform a preproprotein	0.95	**0.76**
56204970	A8YXX4	glutamate-ammonia ligase (glutamine synthase)	0.96	**0.26**
47496673	Q6ICN0	GRB2	1.10	**0.72**
55958754	PHP14	RP11-216L13.10	1.19	**0.44**
55960176	LAMC1	laminin, gamma 1 (formerly LAMB2)	1.22	**0.42**
5679449		OTTHUMP00000016179		**0.69**
34535789	Q6ZR81	unnamed protein product		**0.71**

a Protein is included if at least half of the biologic z-score values are ≥1.960σ (indicated by bolding) and there are no major disagreements between biological replicates.

b L/H ratio refers to the geometric mean of all log_2_ L/H values for each given gi number, expressed as relative protein quantity in infected cultures.

### Validation of SILAC results by Western Blotting

**Figure 4:**
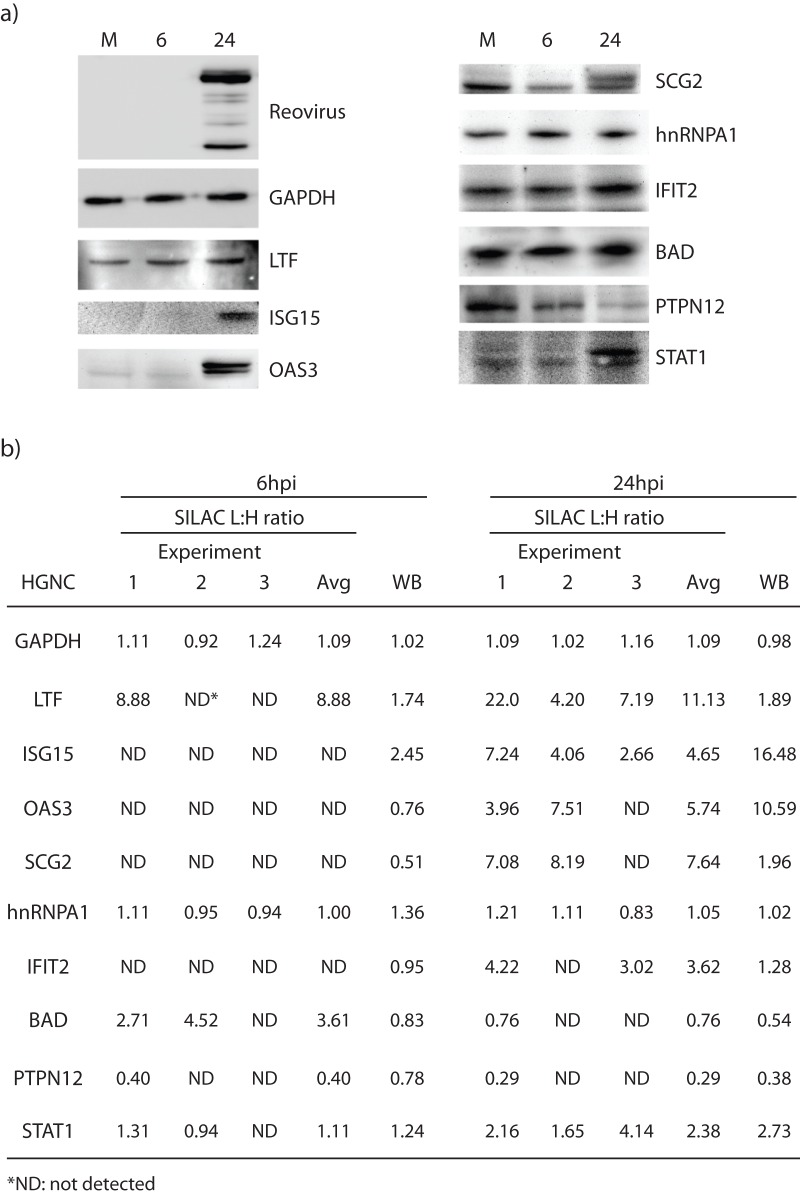
Validation of SILAC-determined protein abundances. (a) Western blot analyses of selected proteins. Mock-infected and T1L-infected cells were harvested at 24hpi, lysed with 0.5% NP-40, and 20–80 μg of each cytosolic fraction resolved in each lane of 10×6.5×0.1 cm 10% SDS-mini-PAGE. Proteins were transferred to PVDF membranes, blocked, probed with various indicated primary antibodies, developed with appropriate secondary antibodies, and visualized with an Alpha Innotech FluorChemQ MultiImage III instrument. (b) Densitometry analysis comparison to SILAC L:H average ratios for the ten different host proteins in (a). Most of the Western blot results correlated to the regulation of the proteins observed in SILAC (whether or not they are up or down regulated). All of the proteins tested by Western blot were also identified at 24hpi in SILAC and were therefore used for confirmation. Some of these proteins were also identified at 6hpi in SILAC and these were used for WB comparison. (GAPDH – glyceraldehyde-3-phosphate dehydrogenase, LTF – lactotransferrin, ISG15 – interferon-stimulated protein 15kDa, OAS3 – 2′–5′oligoadenylate synthetase 3, SCG2 – secretogranin II, hnRNPA1 – heterogeneous nuclear ribonucleoprotein A1, IFIT2 – Interferon-induced protein with tetratricopeptide repeats 2, BAD – Bcl-2 binding component 6, PTPN12 – protein tyrosine phosphatase, non-receptor type 12, STAT1 – signal transducer and activator of transcription 1).

Several of the SILAC-identified up-regulated and non-regulated proteins were examined by Western blotting (Figure 4) and most Western blot results confirmed the trends of the SILAC-determined regulation status, including LTF, ISG15, OAS3, SCG2, PTPN12 and STAT1. GAPDH, which is usually used as a loading control in Western blots, was seen at a 1∶1 infected to mock ratio in all three experiments at both the 6 and 24 hour infection time points, and had SILAC ratios of 1.09±0.11 (Figure 4b). HnRNPA1, another protein shown by SILAC not to be differentially regulated, with ratios of 1.02±0.14, was seen in Western blot with ratios of 1.36 and 1.02 at 6 and 24hpi, respectively. We also used a reovirus antibody to confirm cell infection, which showed up strongly by the later time point. Lactotransferrin was slightly up-regulated at both 6 and 24hpi (1.74 and 1.89, respectively) using Western blot, but SILAC suggested a large up-regulation (8.88 and 11.13, respectively); this may indicate FBS contamination. IFIT2 was indicated in the SILAC experiments to be up-regulated at 24hpi but Western blot suggested a ratio of 1.28. BAD was observed up-regulated >2.5 fold at 6hpi in SILAC, but was only seen at a 0.83 ratio in WB. These few apparent SILAC:WB discrepancies may have been caused by inherent differences in sampling (partially degraded proteins that would not have been measured by Western blot but their peptides would have been detected by MS) or by inherent differences in the different methods' levels of sensitivity.

### Functional and pathway analyses

Lists of proteins observed to be either up- or down-regulated after averaging z-scores from all three biological replicates were uploaded into the DAVID analysis web-based tool [24] (http://david.abcc.ncifcrf.gov/home.jsp) and functional categories were obtained from both GO and Panther terms for both time points. We obtained metabolic and biological functions that are enriched for each list of proteins uploaded at 95, 99, and 99.9% confidence cutoffs respectively. Supplementary Figure S1 shows a list of significantly altered GO biological processes and molecular functions that are plotted against their respective fold enrichment values for each time point. At 6hpi, the top functions that were most affected by the differentially regulated proteins included transforming growth factor β-receptor, activation of pro-apoptotic gene products, cis-trans isomerase activity, late endosome functions and serine protease inhibitor processes (Sup Fig. S1 A, C). At 24hpi, top biological processes included positive regulation of interferon-alpha production, defense response to virus by host, interferon-mediated immunity and procollagen-lysine 5-dioxygenase activity (Sup Fig. S1 B, D). Further analyses were performed using the 95% cutoff, since the more stringent cutoffs identified fewer pathways with no apparent alterations in the top functional networks identified.

**Figure 5:**
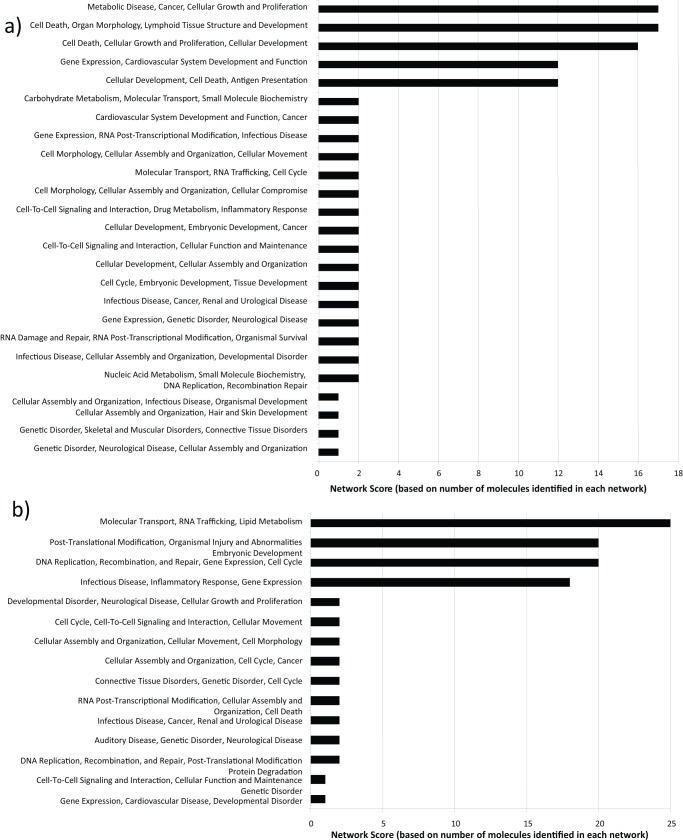
Top network functions generated using Ingenuity protein analysis for HEK293 cells infected with T1L reovirus at (a) 6hpi and (b) 24hpi. Graphs represent host cell functions with highest score (x-axis) based on the number of differentially regulated proteins observed in that network. The higher the score, the greater the number of proteins differentially regulated in that particular function network.

**Figure 6:**
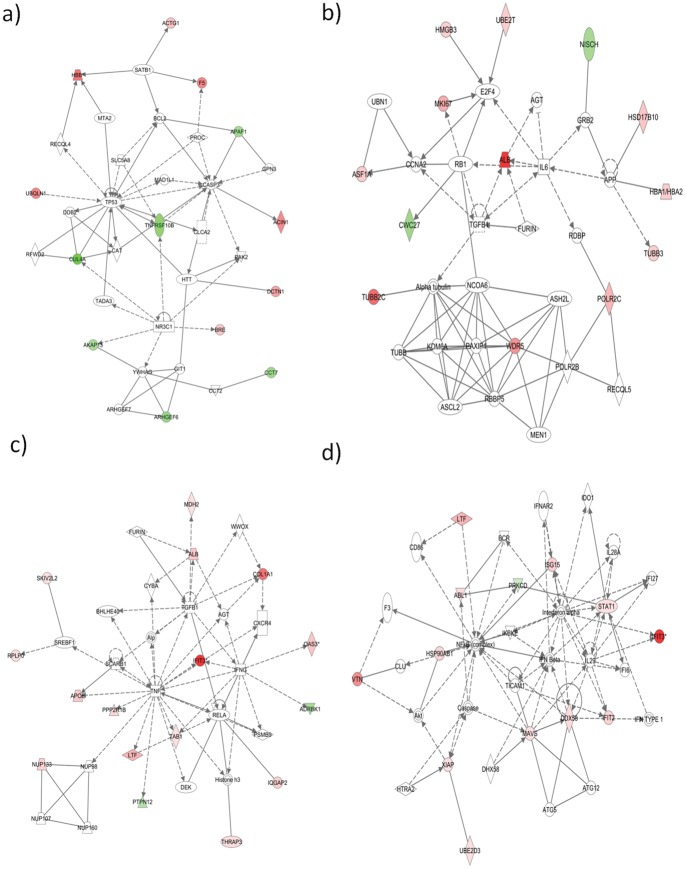
Functional network analysis of differentially regulated proteins observed in T1L infected HEK293 cells at 6hpi (a, b) and 24hpi (c, d). The top network functions identified in the previous figure are shown in more detail with interconnecting protein relationships indicated by solid (direct interaction) or dashed (indirect interaction) lines. Red/pink molecules represent proteins up-regulated; green molecules represent down-regulation. (a) Network 1, 6hpi; top functions include cell death (b) Network 2, 6hpi; top functions include cellular growth and proliferation. (c) Network 1, 24hpi; top functions include molecular transport. (d) Network 4, 24hpi; top functions include infectious disease and inflammatory response.

Proteins were also analyzed by using Ingenuity Pathways Analysis (Ingenuity® Systems, www.ingenuity.com) to generate metabolic and canonical pathways and interconnecting proteins. The top network functions for each of the three separate experiments at 95% z-score cutoff were inflammatory disease, cellular development and tissue development, respectively. To take into account the diversity in identified proteins among the three separate biological replicates, average z-scores were used for further analysis (Figure 5). The top network functions identified as having the most differentially regulated proteins at 6hpi were cell death, cellular growth and proliferation, antigen presentation and gene expression (Figure 6 a, b). A similar analysis was performed for the 24hpi time point. The top networks generated for each of the separate biological experiments were molecular transport, DNA replication and repair, post translational modification and inflammatory response and infectious disease (Figure 6 c, d). This indicates that the host cell proteome changes, signifying host-virus interactions are continuously shifting depending on the stage of the virus life cycle.

**Figure 7:**
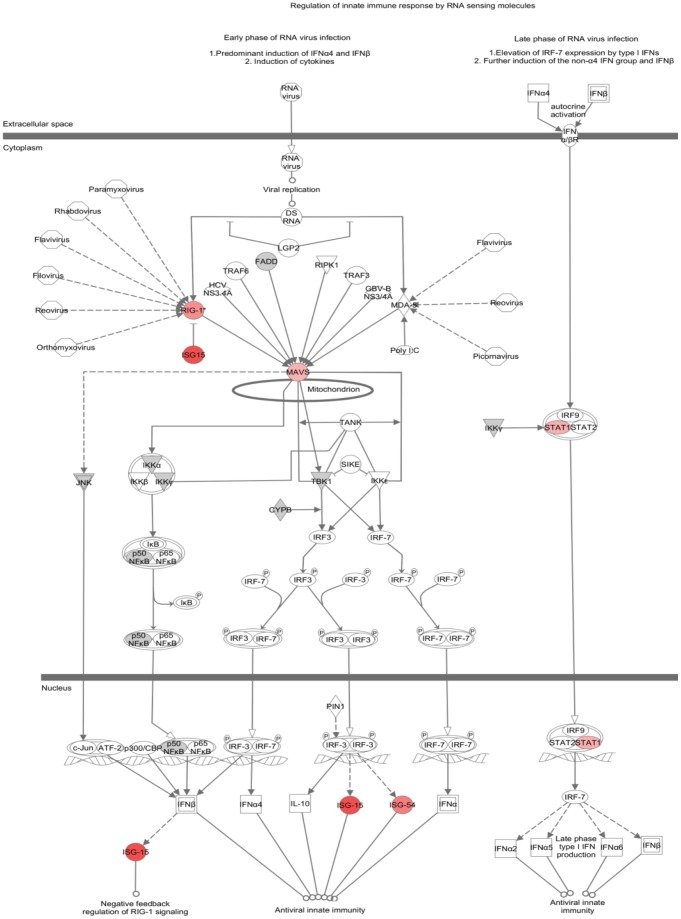
Canonical pathway “Activation of IRF by cytosolic pattern recognition receptors” that was identified as significantly (p-value of 3.7×10^−5^) altered after 24 h of T1L reovirus infection in HEK293 cells. Protein regulation expression patterns overlaid from first biological replicate. Red indicates up-regulation, grey represents proteins not changed in abundance after infection and white represents molecules not identified in SILAC experiment but are part of the known canonical pathway.

Metabolic and cellular canonical signaling pathways were also used for analysis of uploaded datasets. Activation of IRF by cytosolic pattern recognition receptors is one such signaling pathway that was significantly altered by 24hpi. This pathway had an enrichment p-value of 3.7×10^−5^ based on the average z-score analysis at 24hpi. Figure 7 shows this pathway with overlaid expression values from the first biological replicate, which was representative of other replicates for this particular pathway. A number of proteins were identified by SILAC, and many were up-regulated, including RIG-1, ISG-15, MAVS (also known as interferon-β promoter stimulator), STAT1 and ISG-54 (also known as IFIT2). The proteins seen up-regulated in this canonical pathway are those having a role in the early phase of virus infection, functioning in antiviral innate immunity.

## Discussion

SILAC is an effective way to observe differences in the relative protein concentrations of thousands of proteins that may differ in expression levels between two separate experimental cell cultures. Our study quantified changes in the physiological levels of several thousand cellular proteins during T1L infection. We analyzed all data from all three experiments, both separately and in combination, by IPA and DAVID analysis to identify a large list of cell metabolic and biological pathways that were affected by MRV T1L infection. This approach was also used to confirm inter-experiment reproducibility. Comparing the top network (functions in molecular transport, RNA trafficking and lipid metabolism) generated after 24hpi from one experiment to another showed a significant up regulation of the whole network, with numerous proteins up regulated in more than one experiment (Supplemental Figure S2). By studying the difference in networks generated from multiple time points, we examined how the host cell's response to infection changed during the virus life cycle. At the earlier time point, cell death and proliferation as well as antigen presentation are seen as major pathways affected which correlated with DAVID analysis that indicated apoptosis was influenced. At 24hpi, molecular transport, DNA replication and repair, post-translational modification and inflammatory response are more prominently affected, correlating with positive regulation of interferon-alpha production and defense response to virus identified with DAVID. In general, the host response to viral infection produced more up regulated proteins than down regulated ones. Detection of more up regulated proteins than down regulated ones correlates with a previous genomic assay performed on T1L in HEK293 cells that observed this same trend in differential regulation of mRNA [21].

### Comparisons to previous findings

Differentially regulated apoptosis and DNA repair pathway mRNA is observed after infection with T1L and T3D by 24hpi [21]. We also observed differentially regulated proteins in the apoptotic pathway after 6 h and DNA repair after 24 h. Previous studies reported differences in the genomic response to reovirus T3D for proteins such as STAT-1, ISG-type and heat shock proteins [21], [23], [33], [34] and we also observed differential regulation of these types of proteins in our assessment of T1L infection. A proteomic study using four reovirus strains, including T1L, T3D and reassortants identified heat shock proteins, interferon-induced proteins and serine-threonine protein phosphatase as regulated in murine myocytes [23]. We also found the hsp and INF-induced proteins were up-regulated and serine-threonine protein phosphatase protein was down-regulated in our study.

Known canonical pathways were also examined and activation of IRF by cytosolic pattern recognition receptors pathway was shown to be significantly altered (p value 3.7×10^−5^) after 24 h indicating this pathway is important in reovirus infection. Up-regulated proteins in this pathway are RIG-1, ISG15, MAVS, STAT1 and ISG-54 (also known as IFIT2). ISG15 has not previously been identified as affected by MRV infections, but has been identified in numerous other viral infections [35], [36]. As well, numerous other ISG-like proteins have been observed for reovirus [34]. ISG15 plays an important role in antiviral response, and is strongly induced by the type 1 interferon system [37]. In particular, ISG15 production is mediated by the JAK/STAT pathway. IFIT2 RNA expression is induced following infection with MRV strain T3D [34], [38]. Our study showed that the IFIT2 protein was up-regulated following infection with T1L, which may indicate that IFIT2 RNA expression is also induced after T1L infection. IFIT2 is part of a family of proteins that are normally not present in the cell, and are strongly expressed by stimuli such as viral infections. These proteins mediate numerous cell pathways involved in viral functions such as replication, dsRNA signaling, and translational initiation [39]. STAT1, up-regulated in our study, is a commonly activated antiviral protein induced by many viruses, including MRV serotype 3 strain Abney (T3A) [36], [40]. This activation is accomplished by the phosphorylation of STAT1. RIG-1 and MAVS (also known as interferon-β promoter stimulator 1) are required for IRF-3 activation and apoptosis induction in MRV strain type 3 Dearing infections, where rapid apoptosis in cardiac myocytes may play a protective role in infected cells by prohibiting virus spread [38].

### Numerous proteins involved in apoptosis and oncolysis were differentially regulated

In addition to RIG-1 and MAVS, numerous other proteins involved in apoptosis were identified as differentially regulated. A major hub at 6hpi in network 1 (Figure 6a) is the tumor suppressor protein p53, which plays an important role in either the regulation of cell metabolism or apoptosis depending on the stress level of a cell [41]. As indicated earlier, reoviruses are currently being explored as oncolytic therapy because the virus preferentially replicates in cells with abnormal Ras-signaling pathways and induces apoptosis significantly more in these cells [42]. However, the possible role of MRV-induced p53-mediated oncolysis is only partially understood [42]. All reovirus types show oncolytic properties but most work has been performed on MRV strain T3D [17]–[20], [43]. There are differences in the pathogenesis of the different strains of MRV. These differences may give insight into mechanisms of disease as well as oncolytic activity, if different strains are studied. Although p53 itself was not identified in our SILAC analysis, pathway analysis showed a significant interaction network involving this protein. The protein identified as TNFRSF10B, also known as DR5, interacts with p53 to induce apoptosis [44]. DR5 is a component of TRAIL-mediated apoptosis, and is required for reovirus-induced apoptosis in HEK293 cells [45]. TRAIL-mediated apoptosis is marked by an increase in cell surface DR5 expression, peaking at 24hpi [45]. We found DR5 was down-regulated at 6hpi, indicating either a delay in apoptosis, or a change in cell localization out of the cytosol. Two other proteins that interact with p53 are ubiquilin 1 (UBQLN1) and cullin 4A (CUL4A), which were up-regulated and down-regulated, respectively. In HeLa cells, ubiquilin 1 decreases degradation of p53 [46] and cullin 4A increases degradation of p53 in HEK293 cells [47], therefore the combined effect in T1L infection indicates that p53 is being stabilized.

Another hub at 6hpi in network 2 (Figure 6b) is of interest with respect to MRV and cancer therapy. E2F transcription factor 4 normally functions in cell cycle checkpoints, specifically important for entering mitosis. When DNA damage occurs, E2F4 can prevent mitotic entry by down-regulating many targets inducing cell cycle arrest [48]. This protein plays a role in other viral diseases such as SIV encephalitis [49] and is a target of virus protein interactions in Epstein-Barr infections [50]. Although E2F4 itself was not identified in our SILAC results, network analysis illustrated that three proteins recently shown to bind E2F4 [51], namely HMGB3, UBE2T and MKI67, were all up regulated at 6hpi. All three proteins have oncogenic properties [52], [53] and might be of interest concerning the therapeutic use of reoviruses in cancer treatment studies.

### SILAC identifies potentially novel important host regulatory proteins

The top differentially regulated network identified at 24hpi had functions in molecular transport, with TNF alpha as a major hub (Figure 6c). TNF is a multifunctional proinflammatory cytokine, involved in numerous diseases and infections. Our SILAC results did not identify TNF itself, but identified six other proteins that interact with TNF, namely APOE, PPP2R1B, LTF, PTPN12, TAB1 and IFIT3. We are not aware of any of these proteins being previously associated with MRV infections. However, all except PTPN12 are associated with other viral infections. Lactotransferrin/lactoferrin (LTF) and IFIT3, both induced by TNF and up-regulated by reovirus at 24hpi, function in antiviral activities [54], [55]. Apopoliprotein E (APOE) is another mulitifunctional protein that has different effects on virus infection depending on the virus. For example, APOE (isoform 4) is protective against some infections, such as chronic hepatitis C, but allows faster disease progression for HIV, and worse disease symptoms for herpes simplex latencies [56]. Conversely, APOE is essential for initial HCV infectivity [57]. TNF alpha also increases the expression of protein phosphatase 2 (PPP2R1B), which is part of a highly regulated phosphatase family functioning in multiple intracellular pathways, that also happen to be widely targeted by numerous viruses [58]. TAB1, when bound to TAK1, is activated by TNF alpha, and related to the JNK pathway [59], [60]. One of the major roles of TAK1 is to mediate intracellular actions of proinflammatory cytokines [60]. The only protein identified in the TNF alpha hub that was down-regulated, namely PTPN12, is also the only protein that has not yet been reported in any other viral infections to date. Protein tyrosine phosphatases, of which PTPN12 is a member, regulate numerous cellular functions by selective dephosporylation, and dysregulation of these proteins is associated with many diseases [61]. PTPN12 in particular has been recently shown as a tumor suppressor [62], [63]. Because this protein was down-regulated at 24 hpi by reovirus, it may be of particular interest in the oncolytic properties of reoviruses currently being studied. OAS3, identified in Network 1 at 24hpi, may also have an important role in T1L infection, since it has antiviral properties in other viral infections, such as dengue, tick-borne encephalitis and chikungunya virus [64], [65].

In conclusion, we have used SILAC to identify a number of additional novel candidate proteins, more focused study of which should provide greater understanding of MRV-induced pathogenesis as well as a better understanding of how to use this virus as a more effective research tool.

## Supporting Information

Figure S1 DAVID analysis of up (a, b) or down regulated (c, d) proteins using average z-scores from all three biological replicates. Metabolic and biological functions are shown that are enriched from the differentially regulated protein lists at 95, 99, and 99.9% confidence cutoffs (indicated by blue, red and green bars, respectively). At 6hpi (a, c) top functions included transforming growth factor β-receptor, activation of pro-apoptotic gene products, cis-trans isomerase activity, late endosome functions and serine protease inhibitor processes. At 24hpi (b, d) top biological processes included positive regulation of interferon-alpha production, defense response to virus by host, interferon-mediated immunity and procollagen-lysine 5-dioxygenase activity.
(TIF)

Figure S2 Inter-experiment reproducibility of 95% confidence IPA network analyses. Each of the three biologic replicates of the top 24hpi network; molecular transport, RNA trafficking and lipid metabolism, are depicted (a, b, c). Numerous proteins are up regulated in more than one replicate.
(TIF)

Table S1 Table showing all host proteins observed as up-regulated (at 95% confidence) after infection with T1L reovirus at both 6 and 24 hpi. Proteins at the top of the lists are those identified more than one time; proteins in the bottom half are those identified only once. Table shows the individual protein z-scores found for each of the three biological replicates, as well as the average L:H ratios for all three experiments. A protein is included in this table if at least half of the biologic z-score values are ≥1.960σ (indicated by bolding; at least 95% confidence score) and there are no major disagreements between biological replicates.
(XLS)

Table S2 Table showing all host proteins observed as down-regulated (at 95% confidence) after infection with T1L reovirus for both 6 and 24 hpi. Proteins at the top of the lists are those identified more than one time; proteins in the bottom half are those identified only once. Table shows the individual protein z-scores found for each of the three biological replicates, as well as the average L:H ratios for all three experiments. A protein is included in this table if at least half of the biologic z-score values are ≥1.960σ (indicated by bolding; at least 95% confidence score) and there are no major disagreements between biological replicates.
(XLS)
