# Supplementary figures and images for: Quantification of the Host Response Proteome after Mammalian Reovirus T1L Infection
Source: PLoS One. 2012 Dec 11;7(12):e51939. doi: 10.1371/journal.pone.0051939 (PMC3519901; doi:10.1371/journal.pone.0051939)

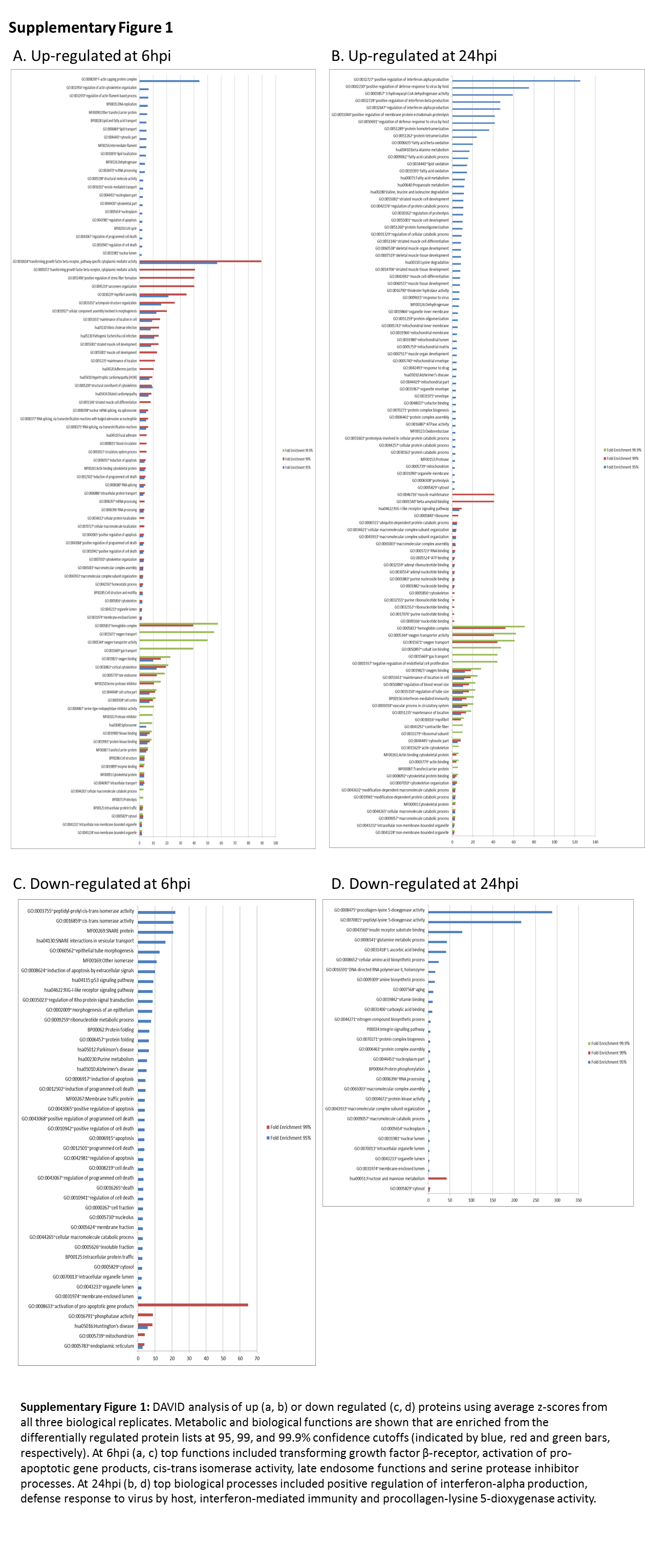

Supplement: Figure S1 — DAVID analysis of up (a, b) or down regulated (c, d) proteins using average z-scores from all three biological replicates. Metabolic and biological functions are shown that are enriched from the differentially regulated protein lists at 95, 99, and 99.9% confidence cutoffs (indicated by blue, red and green bars, respectively). At 6hpi (a, c) top functions included transforming growth factor β-receptor, activation of pro-apoptotic gene products, cis-trans isomerase activity, late endosome functions and serine protease inhibitor processes. At 24hpi (b, d) top biological processes included positive regulation of interferon-alpha production, defense response to virus by host, interferon-mediated immunity and procollagen-lysine 5-dioxygenase activity. (TIF) [file pone.0051939.s001.tif]

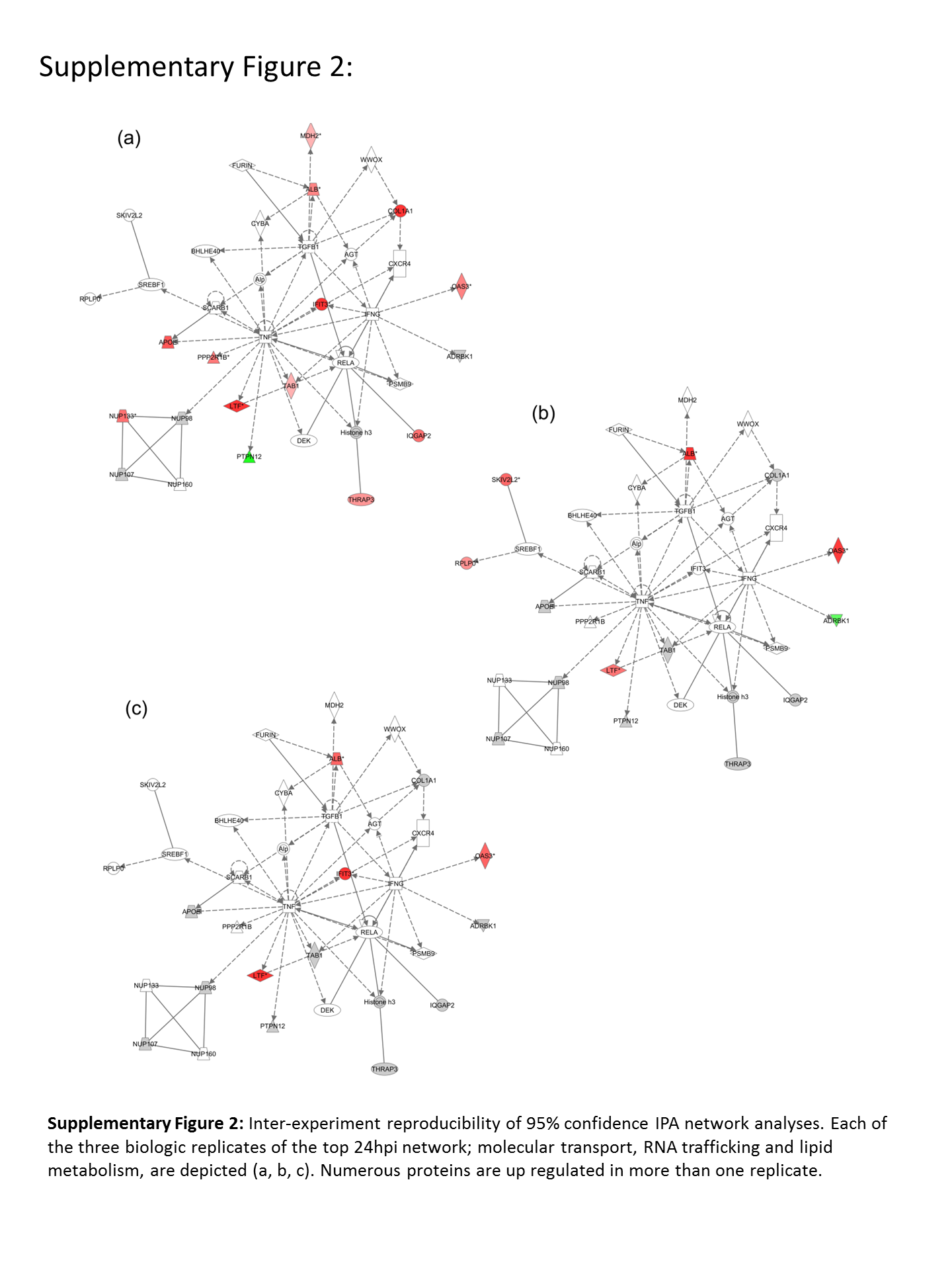

Supplement: Figure S2 — Inter-experiment reproducibility of 95% confidence IPA network analyses. Each of the three biologic replicates of the top 24hpi network; molecular transport, RNA trafficking and lipid metabolism, are depicted (a, b, c). Numerous proteins are up regulated in more than one replicate. (TIF) [file pone.0051939.s002.tif]
